# Salutation

**Published:** 1903-01

**Authors:** 


### Editorial.

### SALUTATION.

WITH this number of the INTERNATIONAL DENTAL JOURNAL commences the fourteenth year under its present auspices. Those who have preserved their copies during this period will find it contains a large mass of very good and useful information upon subjects both scientific and practical. Its contents during those years will compare favorably with any other of the dental journals published during the same time.

The present plans for the INTERNATIONAL DENTAL JOURNAL include a general increase of its pages, a regularly appearing review of the important articles in current dental journals, an enlargement of well-selected miscellany, composed of practical matter. Endeavors to secure a greater number of papers upon subjects related to practice will be made, and appeals to its readers and to the associations it represents to contribute to it short articles in elucidation of technical subjects.

The INTERNATIONAL DENTAL JOURNAL was started and has been maintained on the ground that a strictly professional periodical should exist in which there should be no divided interests. It has aimed to avoid the paternalism which, however pleasant to many, is inconsistent with manly professional life and aspirations. It was felt that the deadening influences upon the profession could be best counteracted by the support of a class of journalism which would avoid the commitment of those desiring an independent effort to the custody of those whose animating motive is the cultivation of their business interests. There was no question that in the earlier days of the dental profession the trade journals were of great benefit, and, the leading ones continuing to render good service, the need of this kind of paternalism had well passed. It was considered by many that the times had ripened for the commencement and development of a journal that would tend to create a higher grade of professional sentiment and cultivate a feeling of solidarity among dentists the country over. This sentiment was founded in a sense of right and honor. For the defence of this right and principle this journal stands.

It is most particularly pertinent, in view of the fact that all of the trade journals have so clearly made it apparent by their recent action that trade influences are paramount with them, that the INTERNATIONAL DENTAL JOURNAL should reaffirm the motive for its existence. It appeals to professional spirit to give it renewed and *extended* support.

Our position can be best understood by the feeling which would arise among a religious body were its literature gathered and published by a department store, or a syndicate of undertakers; or the medical profession, were its magazines generally under the control of makers of surgical instruments or purveyors of medical supplies; or the Society of Ophthalmologists dependent upon an enterprising optician; or the legal profession, if it were under the paternalism of a vender of legal books.

The dental profession certainly has not become so deeply somnolent that it cannot perceive that a better state than is indicated above is desirable.

